# COVID-19 epidemic phases and morbidity in different areas of Chinese mainland, 2020

**DOI:** 10.3389/fpubh.2023.1151038

**Published:** 2023-04-06

**Authors:** Yuehai Wang, Jinghong Yu, Fei Wang, Yuqiang Zhang, Shengjun Ma

**Affiliations:** ^1^Heart Center and Laboratory Animal Center, Liaocheng People's Hospital of Shandong First Medical University, Liaocheng, Shandong, China; ^2^Hospital Library, Liaocheng People's Hospital, Liaocheng, Shandong, China; ^3^Department of Cardiology, Shandong Corps Hospital of Chinese People's Armed Police Forces, Jinan, China

**Keywords:** COVID-19, Chinese mainland, epidemic, prevention, isolation measures

## Abstract

**Background:**

In the early stage of COVID-19 epidemic, the Chinese mainland once effectively controlled the epidemic, but COVID-19 eventually spread faster and faster in the world. The purpose of this study is to clarify the differences in the epidemic data of COVID-19 in different areas and phases in Chinese mainland in 2020, and to analyze the possible factors affecting the occurrence and development of the epidemic.

**Methods:**

We divided the Chinese mainland into areas I, I and III, and divided the epidemic process into phases I to IV: limited cases, accelerated increase, decelerated increase and containment phases. We also combined phases II and III as outbreak phase. The epidemic data included the duration of different phases, the numbers of confirmed cases, asymptomatic infections, and the proportion of imported cases from abroad.

**Results:**

In area I, II and III, only area I has a Phase I, and the Phase II and III of area I are longer. In Phase IV, there is a 17-day case clearing period in area I, while that in area II and III are 2 and 0 days, respectively. In phase III or the whole outbreak phase, the average daily increase of confirmed cases in area I was higher than that in areas II and III (*P* = 0.009 and *P* = 0.001 in phase III; *P* = 0.034 and *P* = 0.002 in the whole outbreak phase), and the average daily in-hospital cases were most in area I and least in area III (*P* = 0.000, *P* = 0.000, and *P* = 0.000 in phase III; *P* = 0.000, *P* = 0.000, and *P* = 0.009 in the whole outbreak phase). The average number of daily in-hospital COVID-19 cases in phase III was more than that in phase II in each area (*P* = 0.000, *P* = 0.000, and *P* = 0.001). In phase IV, from March 18, 2020 to January 1, 2021, the increase of confirmed cases in area III was higher than areas I and II (both *P* = 0.000), and the imported cases from abroad in Chinese mainland accounted for more than 55–61%. From June 16 to July 2, 2020, the number of new asymptomatic infections in area III was higher than that in area II (*P* = *0.000*), while there was zero in area I. From July 3, 2020 to January 1, 2021, the increased COVID-19 cases in area III were 3534, while only 14 and 0, respectively, in areas I and II.

**Conclusions:**

The worst epidemic areas in Chinese mainland before March 18, 2020 and after June 15, 2020 were area I and area III, respectively, and area III had become the main battlefield for Chinese mainland to fight against imported epidemic since March 18, 2020. In Wuhan, human COVID-19 infection might occur before December 8, 2019, while the outbreak might occur before January 16 or even 10, 2020. Insufficient understanding of COVID-19 hindered the implementation of early effective isolation measures, leading to COVID-19 outbreak in Wuhan, and strict isolation measures were effective in controlling the epidemic. The import of foreign COVID-19 cases has made it difficult to control the epidemic of area III. When humans are once again faced with potentially infectious new diseases, it is appropriate to first and foremost take strict quarantine measures as soon as possible, and mutual cooperation between regions should be explored to combat the epidemic.

## Introduction

Coronavirus disease 2019 (COVID-19) was first reported in Wuhan city of China at the end of 2019 (1). The gene sequence of the virus was first identified and published in China (2). After its human-to-human transmission route was identified, COVID-19 was classified as a Class B infectious disease and managed according to Class A infectious disease (3). During the outbreak period in Wuhan, the maximum number of confirmed COVID-19 cases per day was 1,985, and the maximum number of in-hospital COVID-19 cases per day was 38,020. The COVID-19 cases and close contacts were strictly isolated, the residential areas in Wuhan were under closed management, the vehicles were stopped, and the traffic between Wuhan and the outside world was closed (4–7). And the epidemic was once effectively controlled in Wuhan and even in the whole Chinese mainland, but COVID-19 has spread faster and faster worldwide, affecting 228 countries and territories (8). The emergence of COVID-19 variants Delta and Omicron (9), especially Omicron, has inevitably increased the difficulty of combating the epidemic. However, the virus mutation will not stop, and its transmission ability and lethality are also changing. In the face of the mutant virus, the effectiveness of the vaccine needs to be further observed. And mankind continues to face constant challenges.

The emergence and outbreak of COVID-19 epidemic in Chinese mainland is still a hot research topic. We assume that Chinese mainland has both valuable measures and shortcomings in the initial stage of combating the epidemic. The purpose of our research is to explore various factors that affect the occurrence and development of the COVID-19, and remind people to avoid detours when fighting against the epidemic (including unknown infectious diseases) in the future. To facilitate understanding of the epidemic in Chinese mainland, 2020, we divided Chinese mainland into three adjacent areas around Wuhan for the first time in the world, and divided the evolution of the pandemic into four phases. We tracked and compared the epidemic data in different phases and areas, including the data of confirmed, hospitalized cases and asymptomatic infections, as well as the data of imported cases from abroad. In addition, this article also covers the lack of understanding of the transmission route and speed of the virus when Wuhan first encountered this new virus, and the subsequent isolation measures.

## Methods

**Figure 1:**
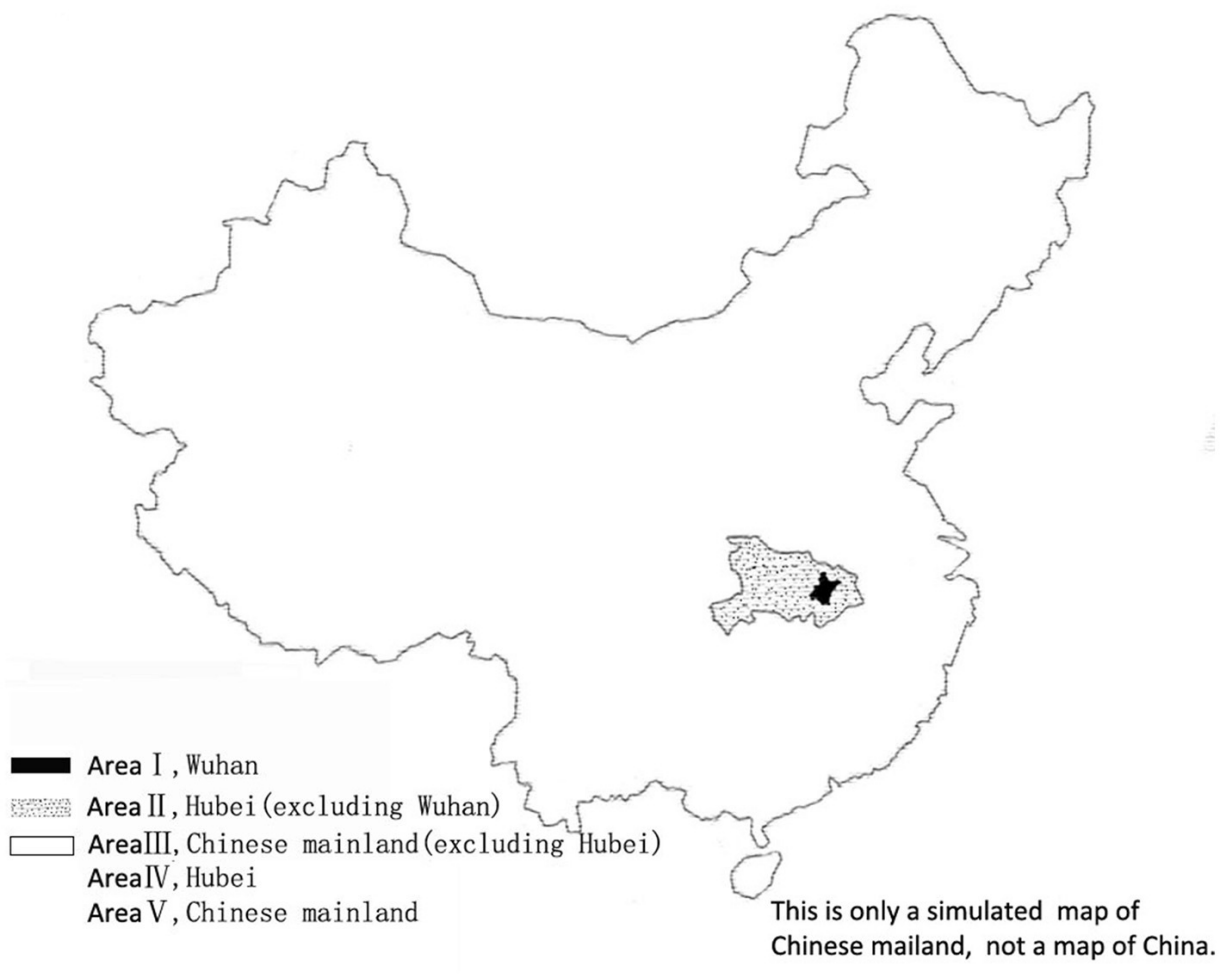
Division of COVID-19 epidemic areas in Chinese mainland.

In Chinese mainland, Wuhan was the first place where COVID-19 cases were found and infectious diseases broke out. According to the borders of Wuhan City, Hubei Province and Chinese mainland, we divided Chinese mainland into three adjacent areas: area I (Wuhan city), area II (Hubei province, excluding Wuhan city) and area III (Chinese mainland, excluding Hubei province) (Figure 1), centering around Wuhan. In addition, we called Hubei province (including Wuhan city) area IV, and called Chinese mainland area V (including Hubei province) (Figure 1). All medical and government departments have participated in the collection of epidemic data, and the state does not allow the omission of epidemic data. Health committees at all levels published the newly collected data in their jurisdiction every day. We tracked the daily epidemiological data of COVID-19 from the official websites of health committees at all levels before January 1, 2021. The epidemic data include the numbers of confirmed COVID-19 cases, asymptomatic COVID-19 infections and imported COVID-19 cases from abroad, and the ratio of imported COVID-19 cases from abroad. Cases with epidemiological history, clinical manifestations of fever or upper respiratory tract infection, imaging features of pneumonia, normal or decreased total white blood cells, or decreased lymphocyte count in the early stage of the disease, COVID-19 nucleic acid positive or gene highly homologous or specific IgM and IgG antibody positive, or the specific IgG antibody changing from negative to positive or the IgG antibody titer increasing by 4 times or more in the recovery phase than in the acute phase, are defined as confirmed COVID-19 cases (10–13). No clinical symptoms, respiratory tract and other samples with positive COVID-19 or positive serum specific IgM antibody are defined as asymptomatic COVID-19 infections (14).

According to the change trend of the number of confirmed COVID-19 cases, we divided the epidemic process into four phases: limited cases phase (Phase I) (Supplementary material 1, Figure 2A), the number of confirmed COVID-19 cases remained unchanged within a few days after the first diagnosis by nucleic acid test; Outbreak phase (Phase II and III), the number of confirmed COVID-19 cases increased rapidly after phase I, and the daily case-growth accelerated before reaching the peak and then decelerated. Therefore, the outbreak phase was divided into accelerated and decelerated increase phases, those were phase II and III (Supplementary material 1, Figure 2A); Containment phase (Phase IV), after phase III, the trend of daily growth of confirmed COVID-19 cases was almost zero, or tended to zero or there was no trend of repeated outbreak (Supplementary material 1, Figure 2B). According to the aforementioned rule of phases division, the epidemic process of areas I was divided into phase I, II, III, and IV, while the epidemic process of area II and III lacked phase I (Supplementary material 1, Figures 2A, B). In addition, the epidemic processes of area IV and V were divided into phase I, II, III, and IV, respectively (Supplementary material 1, Figures 2A, B).

**Figure 2:**
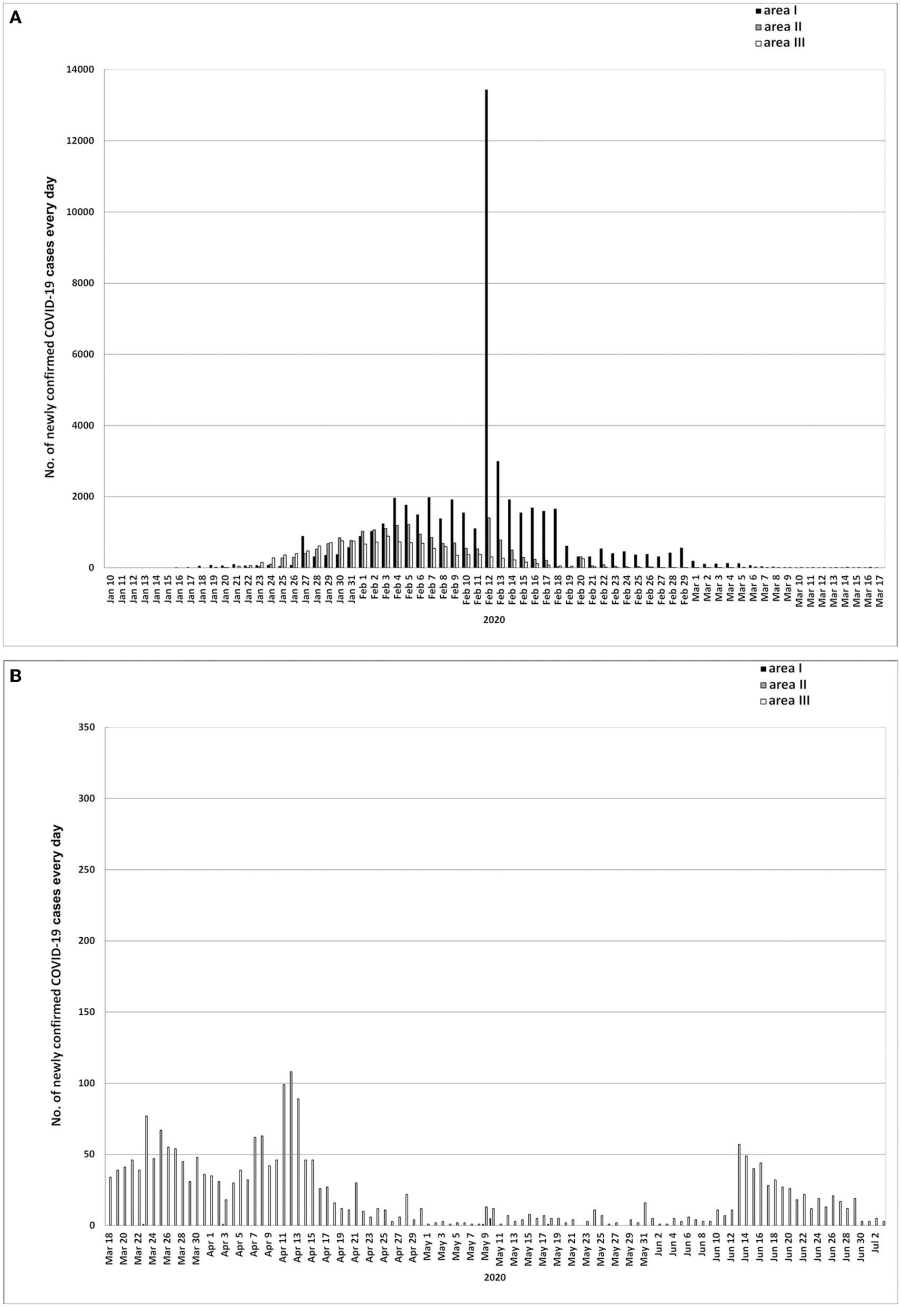
**(A, B)** Daily increase of confirmed COVID-19 cases in areas I, II, and III. Area I, Wuhan city. Area II, Hubei province (excluding Wuhan city). Area III, Chinese mainland (excluding Hubei province). From January 12, 2020, the sudden increase of new confirmed cases are related to the clinical cases (Nucleic acid undetected) from Hubei province (including Wuhan) being classified as confirmed cases. COVID-19 cases do not include asymptomatic COVID-19 infections.

**Figure 3:**
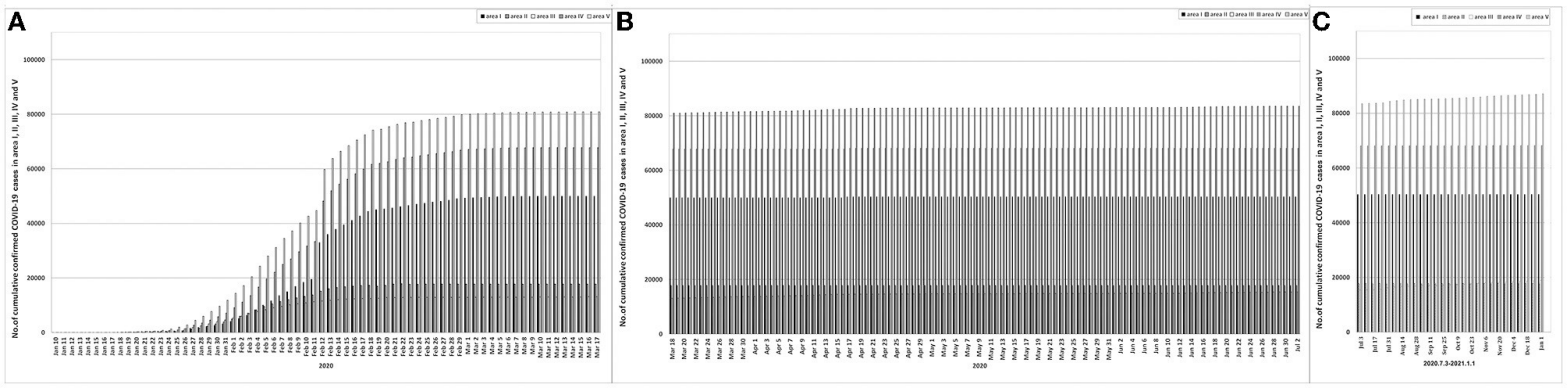
**(A–C)** Cumulative COVID-19 cases updated daily (or weekly) in areas I, II, III, IV, and V. The data before July 3, 2020 was updated once a day, and the data after July 3, 2020 was updated once a week. Area I, Wuhan city. Area II, Hubei province (excluding Wuhan city). Area III, Chinese mainland (excluding Hubei province). Area IV, Hubei province (including Wuhan city). Area V, Chinese mainland (including Hubei province). On January 12, 2020, the sudden increase of confirmed cases was related to the clinical cases (Nucleic acid undetected) from Hubei province (including Wuhan) being classified as confirmed cases. COVID-19 cases do not include asymptomatic COVID-19 infections.

**Figure 4:**
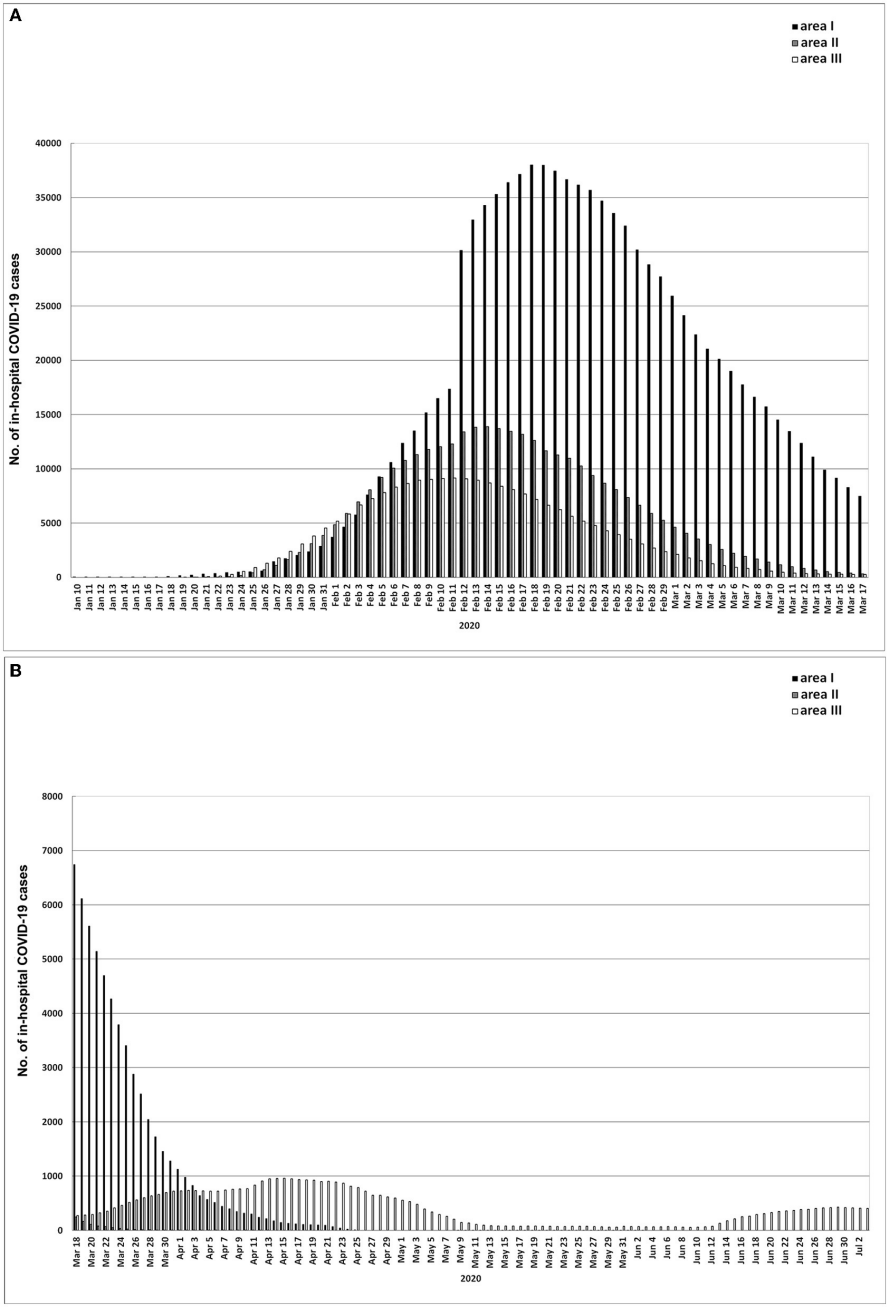
**(A, B)** In-hospital COVID-19 cases updated daily in areas I, II, and III. Area I, Wuhan city. Area II, Hubei province (excluding Wuhan city). Area III, Chinese mainland (excluding Hubei province).

**Figure 5:**
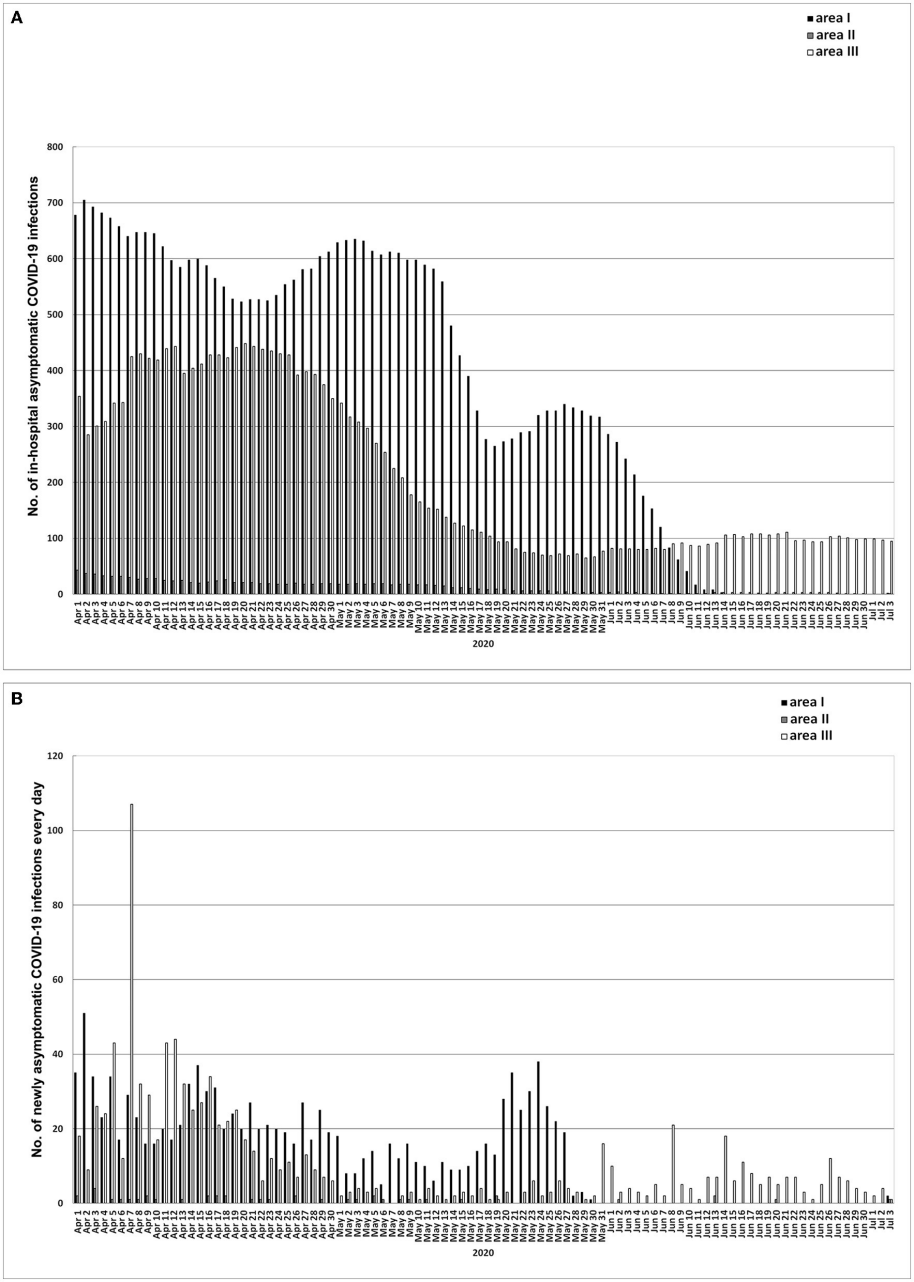
Data of asymptomatic COVID-19 infections. **(A)** In-hospital asymptomatic COVID-19 infections updated daily in areas I, II, and III. **(B)** Daily increase of asymptomatic COVID-19 infections in areas I, II, and III. Area I, Wuhan city. Area II, Hubei province (excluding Wuhan city). Area III, Chinese mainland (excluding Hubei province). The data of asymptomatic COVID-19 infections could be obtained through the websites of health committees at all levels only after March 31, 2020.

**Figure 6:**
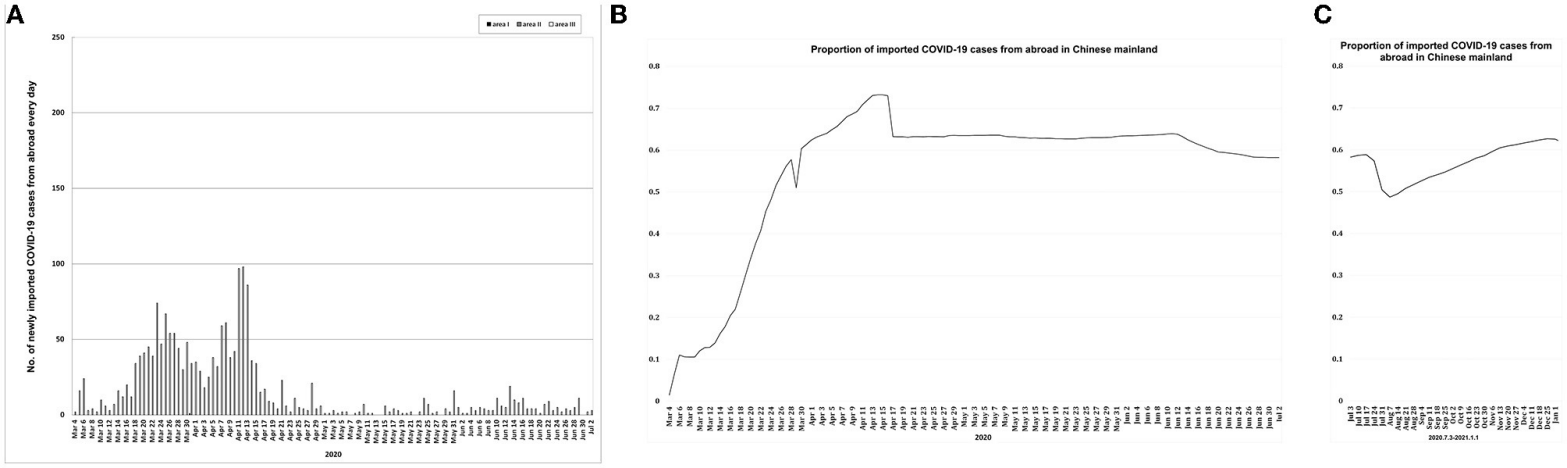
Data of imported COVID-19 cases from abroad. **(A)** Daily increase of imported COVID-19 cases from abroad in areas I, II and III from March 4, 2020 to July 2, 2020. **(B, C)** Proportion of cumulative imported COVID-19 cases from abroad to cumulative COVID-19 cases in Chinese mainland (area V) from March 4, 2020 to January 1, 2021. Area I, Wuhan city. Area II, Hubei province (excluding Wuhan city). Area III, Chinese mainland (excluding Hubei province). The data of imported COVID-19 cases from abroad could be obtained through the websites of health committees at all levels only after March 2 or 3, 2020.

The difference of data among different areas and phases was compared by ANOVA, and was called statistically significant at the 0.05 level. The data was expressed as mean plus minus standard error. SPSS13.0 was used for statistics. We used Excel 97 and Adobe Photoshop CS4 to draw the data into Figures 2–**6**.

In the early stage of the epidemic, the detection capacity of COVID-19 nucleic acid was insufficient. In order to enable COVID-19 cases to receive timely formal treatment and further improve the success rate of treatment, the clinical cases (Nucleic acid undetected) in areas I and II were classified as confirmed cases from January 12, 2020, resulting in a sudden and significant increase of newly confirmed COVID-19 cases (15). In addition, the COVID-19 cases mentioned in the article do not include asymptomatic COVID-19 infections.

## Results

### Time distribution differences of epidemic phases in different areas

Compared with areas II and III, the epidemic of area I alone had a phase I, and had a longer outbreak phase (62 days) (Supplementary material 1, Figures 2A, 3A). Although area I entered the outbreak phase earlier, the containment phase (phase IV) of area I came later because area I had longer phase II and III than areas II and III (Supplementary material 1, Figures 2A, 3A). In addition, the outbreak phase of area II and III started and ended almost at the same time, so they have similar duration: 45 and 48 days, respectively (Supplementary material 1, Figure 2A). After entering phase IV, area I gradually ushered in a 17-day case clearing period, while the case clearing periods of area II and III were only 2 days and zero, respectively (Figure 2).

### Number of confirmed COVID-19 cases and asymptomatic COVID-19 infections in different areas

#### Outbreak phase

During the outbreak phase of areas I, II and III, the cumulative numbers of confirmed cases on the first day, the day when the daily increased cases reached the peak and the last day were 45, 13,603 and 50,005 (area I), 12, 9,548 and 17,795 (area II), and 16, 6,916 and 12,958 (area III), respectively (Figures 2A, 3A). The number peaks of newly diagnosed COVID-19 cases updated daily were 1,985 (area I, February 7), 1,221 (area II, February 5) and 888 (area III, February 3) (Figure 2A). The number peaks of in-hospital COVID-19 cases updated daily were 38,020 (area I, February 18), 13,886 (area II, February 14) and 9,141 (area III, February 11) (Figure 4A). In phase III, even in the whole outbreak phase, the average daily increase of confirmed cases in area I was significantly higher than that in areas II and III (*P* < 0.05) (Supplementary material 2), and the average daily in-hospital cases were most in area I and least in area III among areas I, II, and III (*P* < 0.01) (Supplementary material 3). In addition, in areas I, II, and III, the average number of daily in-hospital COVID-19 cases in phase III was more than that in phase II of the same area (*P* < 0.01) (Supplementary material 3).

### Containment phase

#### March 18 to June 15, 2020

The average daily increase of confirmed cases in area III was higher than that in areas I and II (*P* < 0.01) (Supplementary material 2), and the average daily in-hospital cases were more in areas I and III than in area II (*P* < 0.01) (Supplementary material 3). The daily increase of asymptomatic infections in areas I and III was higher than that in area II (*P* < 0.01) (Supplementary material 4, Figure 5B). In areas I, II, and III, the average daily in-hospital asymptomatic infections were most in area I, followed by area III, and the least in area II (*P* < 0.01) (Supplementary material 5, Figure 5A).

#### June 16 to July 2, 2020 (case clearing period of area I)

In area III, the daily increased confirmed cases and in-hospital COVID-19 cases were 18.88 ± 2.61 and 365.18 ± 13.84, respectively, while in area I or II, both were zero (Supplementary material 2, 3). In area III, the daily increased and in-hospital asymptomatic infections were higher than those in area II (both *P* < 0.01), while in area I, both were zero (Supplementary material 4, 5, Figures 5A, B).

#### July 3, 2020 to January 1, 2021

Cumulative COVID-19 cases in area III increased by about 0.23 times, that is, 3,534 cases (16–19), while only 14 (20, 21) and 0 (16, 19–21) in areas I and II, respectively, in 182 days. The average weekly increased COVID-19 cases in area III were more than those in areas I and II (*P* < 0.01) (Supplementary material 6).

### Imported COVID-19 cases

Before March 4, 2020, Chinese mainland only had accumulated 18 imported COVID-19 cases from abroad (22). After March 17, 2020, the imported cases accounted for more than 50% (Figure 6B). From March 18, 2020 to July 2, 2020, the average proportion of imported cases was 0.612 ± 0.007 in Chinese mainland (area V) (Figure 6B), while there was only one and zero imported COVID-19 case in area I and II, respectively (Figure 6A). From July 3, 2020 to January 1, 2021, the imported COVID-19 cases accounted for more than 55% in Chinese mainland (area V) (Figure 6C).

## Discussion

Wuhan Municipal Health Commission first released the epidemic of viral pneumonia on December 31, 2019 (1). On January 8, 2020, the virus was initially identified as novel coronavirus. On January 10, 2020, after nucleic acid detection, of all previous cases, 41 cases were confirmed as COVID-19 cases (23). Among the confirmed cases, the earliest time for cases to develop symptoms was December 8, 2019 (24). Therefore, the time of human initial infection with COVID-19 in Wuhan may be before December 8, 2019.

Different from areas II and III, only area I (Wuhan City) presented phase I (limited cases phase) of the epidemic, and the reason is unclear. In the early stage of the new epidemic, people knew little about COVID-19 and the pathogen detection technology was insufficient (25, 26), which might lead to missed diagnosis of COVID-19 cases. In addition, it was difficult to avoid underreporting epidemic cases (25). Therefore, the number of COVID-19 cases may be more than 41 during phase I, and we boldly speculate that the phase I of Wuhan epidemic may belong to the phase II in whole or in part. In other words, before January 16 or even 10, 2020, the epidemic of Wuhan might have entered the outbreak phase.

The epidemic of Wuhan (area I) alone had a limited cases phase, and had a longer outbreak phase (62 days) (Supplementary material 1, Figure 2A), which directly determined that the epidemic in the whole Hubei province (area IV) and the whole Chinese mainland (area V) had the same time limited cases phase and outbreak phase as that in area I (Supplementary material 1). Before the end of the outbreak phase, the daily increased cases and in-hospital cases in area I were more than those in areas II and III (Supplementary material 2, 3), which determined the epidemic trend in the whole Hubei Province (area IV) and the whole Chinese mainland (area V) (Figures 3A, 4A). Accordingly, before March 18, 2020, the epidemic situation in Wuhan played a decisive role in the epidemic situation in the whole Chinese mainland.

Wuhan had the most daily increased cases and in-hospital cases, while area III had the least in-hospital cases before March 18, 2020. We know that Wuhan was the place where the epidemic was first discovered. Area II is adjacent to Wuhan, and area III is farther from Wuhan (Figure 1). Spatially, the epidemic is easier to spread in Wuhan than in area II or III. In addition, the outbreak time of areas II and III is later than that of area I, so areas II and III have more time and more powerful measures to prevent COVID-19 outbreak. These may be the reason why the epidemic situation of outbreak phase in areas II and III is less severe than that in area I. At the beginning of the epidemic, Wuhan Municipal Health Commission mentioned several times that no clear evidence of human-to-human transmission was found (1, 23, 27–29), or the possibility of limited human to human transmission could not be ruled out, but the risk of continuous human to human transmission was low (29). Until January 20, 2020, when areas II and III epidemic began to appear, the National Health Commission classified COVID-19 epidemic as a class B infectious disease, and took prevention and control measures for class A infectious diseases to control the COVID-19 epidemic (3). Since then, close contacts could be isolated for medical observation according to law. However, 43 days have passed since the first symptom of the COVID-19 case was diagnosed in Wuhan. Accordingly, in the early stage of the epidemic of Wuhan, the deficient understanding of the transmission route of COVID-19 was easy to weaken people's awareness of prevention, and close contacts could not be isolated according to law for effective medical observation, which made the epidemic easy to spread in Wuhan and might be part of the reason why the outbreak phase epidemic of Wuhan was more severe than that in areas II and III. Therefore, when we again suffer from new infectious diseases with unclear transmission routes, we should first take strict isolation measures, which of course also requires corresponding laws to comply with.

On June 16, 2020, earlier than areas II and III, Wuhan entered a 17-day case clearing period of the epidemic, which not only marked the temporary termination of the epidemic of Wuhan, this also reflected that after the outbreak phase, the epidemic situation in Wuhan had been better controlled than that in areas II and III. In the outbreak phase, in addition to people wearing masks and trying to avoid personnel gathering, Wuhan also implemented measures to stop bus, subway, ferry and network taxi transportation (4), and implemented closed management for all residential areas (5). In the central urban area, motor vehicles were prohibited except for the licensed guaranteed supply of transport vehicles, free transport vehicles and official vehicles (5). From January 23 to April 8, 2020, the measure of closing the roads leaving Wuhan for 76 days was implemented (4, 6, 7). These measures not only limited the spread of the epidemic to areas outside Wuhan, but also effectively curbed the spread of the epidemic within Wuhan. In addition, these measures also avoided the adverse impact of the epidemic outside Wuhan on Wuhan. Therefore, these measures have played an important role in Wuhan entering the case clearing period earlier and ending the epidemic temporarily.

From March 18, 2020 to June 15, 2020, the number of in-hospital asymptomatic infections in area I was still more than that in area III (Supplementary material 5), and no matter the number of in-hospital cases or the number of new asymptomatic infections, there was no statistical difference between area I and III (Supplementary material 3, 4). However, after June 15, 2020, all the data of area III, including newly increased and in-hospital confirmed cases or asymptomatic infections, and newly increased imported cases, were the largest in area I, II and III (Supplementary material 2–6, Figures 2B, 4B, 5A, B, 6A) (16–21). The worst epidemic area in Chinese mainland after June 15, 2020 was area III, which led to the subsequent trend of the epidemic in whole Chinese mainland (Figures 3B, C). The reason might be that area III was adjacent to many countries, had more contacts with the outside world, and was more vulnerable to the epidemic in other countries. This also determined that area III failed to have a case clearing period in phase IV, and directly faced a greater risk of another COVID-19 epidemic outbreak.

From March 18, 2020 to July 2, 2020, almost all the new confirmed cases were in area III (Supplementary material 2, 6, Figure 2). From July 3, 2020 to January 1, 2021, there were newly increased 14, 0, and 3,512 COVID-19 cases in areas I, II, and III respectively (16–21). Before March 4, 2020, Chinese mainland only had accumulated 18 imported COVID-19 cases from abroad (22). But the imported cases from abroad rapidly accounted for more than 55–61% from March 18, 2020 to January 1, 2021 (Figures 6B, C), and almost all the imported COVID-19 cases entered area III (Figure 6A). Accordingly, after March 18, 2020, almost all the increased and imported COVID-19 cases were in area III, and the imported cases accounted for the majority. As a result, since March 18, 2020, area III had become the main battlefields for Chinese mainland to fight against imported epidemic.

In today's world, facing the continuous spread of COVID-19 epidemic, no country can be spared. Epidemic situations in different regions or countries will affect each other. In the process of combating the epidemic, mutual cooperation may be the only choice for mankind. With the improvement of people's awareness of epidemic prevention, their in-depth understanding of COVID-19, the popularity of cheap and effective vaccines and specific drugs, and the decline of virus virulence caused by virus mutation, people will eventually overcome the epidemic, however, we should not forget the disasters brought by COVID-19 to the whole world, and we should not forget the detours and even mistakes we have made in preventing and controlling the epidemic in the world. In the early stage of the epidemic, the virus was highly toxic and the mortality rate was high, and the World Health Organization unremittingly called on people to find COVID-19 cases as soon as possible and implement effective isolation and defense measures as soon as possible without hesitation, which was a valuable recommendation, but many people, regions and countries did not actively and effectively implement it.

In this study, the epidemic data were reported to the health committees at all levels from multiple regions, involving many reporting staff and reporting links. In the process of collecting and transmitting epidemic data, there were inevitably missing reports and false reports. In addition, referring to the boundary of administrative regions, we divide Chinese mainland into area I, II, and III from Wuhan as the center. The area and number of people in the three areas were different.

## Conclusions

In Wuhan, human COVID-19 infection might occur before December 8, 2019, while the outbreak phase might occur before January 16 or even 10, 2020. The worst epidemic areas in Chinese mainland before March 18, 2020 and after June 15, 2020 were area I and area III, respectively, and area III had become the main battlefields for Chinese mainland to fight against imported epidemic since March 18, 2020. Area I alone had a long case clearing period of 17 days in phase IV. Insufficient understanding of COVID-19 hindered the implementation of early effective isolation measures, leading to COVID-19 outbreak in Wuhan, and strict isolation measures were effective in controlling the epidemic. When we again suffer from new infectious diseases with unclear transmission routes, we should first take strict isolation measures, which of course also requires corresponding laws to comply with. The import of foreign COVID-19 cases has made it difficult to control the epidemic of area III. Epidemic situations in different regions or countries will affect each other. In the process of combating the epidemic, mutual cooperation may be the only choice for mankind.

## Data availability statement

The raw data supporting the conclusions of this article will be made available by the authors, without undue reservation.

## Author contributions

All authors listed have made a substantial, direct, and intellectual contribution to the work and approved it for publication.
