# Supplementary material for: COVID-19 epidemic phases and morbidity in different areas of Chinese mainland, 2020
Source: Front Public Health. 2023 Apr 6;11:1151038. doi: 10.3389/fpubh.2023.1151038 (PMC10117903; doi:10.3389/fpubh.2023.1151038)
Supplement: Supplementary file 1 [file Table_1.DOC]

|  | **phase Ⅰ**  (limited cases phase) | **phase Ⅱ**  (accelerated increase phase) | **phase Ⅲ**  (decelerated increase phase) | **phase Ⅳ**  (containment phase) |
| --- | --- | --- | --- | --- |
| **area Ⅰ** | **6** days  (Jan 10 to 15, 2020) | **23**days  (Jan 16 to Feb 7, 2020) | **39** days  (Feb 8 to Mar 17, 2020) | (Mar 18, 2020 to) |
| **area Ⅱ** | **0** day | **17** days  (Jan 20 to Feb 5, 2020) | **28** days  (Feb 6 to Mar 4, 2020) | (Mar 5, 2020 to) |
| **area Ⅲ** | **0** day | **16** days  (Jan 19 to Feb 3, 2020) | **32** days  (Feb 4 to Mar 6, 2020) | (Mar 7, 2020 to) |
| **area IV** | **6** days  (Jan 10 to 15, 2020) | **20** days  (Jan 16 to Feb 4, 2020) | **42** days  (Feb 5 to Mar 17, 2020) | (Mar 18, 2020 to) |
| **area V** | **6** days  (Jan 10 to 15, 2020) | **20** days  (Jan 16 to Feb 4, 2020) | **42** days  (Feb 5 to Mar 17, 2020) | (Mar 18, 2020 to) |

**Supplementary material 1 Division of COVID-19 epidemic phases in Chinese mainland (area I, II and III)** This table covers the period from January 10, 2020 to January 1, 2021. In the division of epidemic phases, we excluded the data of area I and II on February 12 and 13, 2020. The reason is that the data is unusually large, or it is inappropriate to represent the real data of the day. area I, Wuhan. area II, Hubei province (excluding Wuhan city). area III, Chinese mainland (excluding Hubei province). area IV, Hubei province (including Wuhan city). area V, Chinese mainland (including Hubei province).
