# Supplementary material for: COVID-19 epidemic phases and morbidity in different areas of Chinese mainland, 2020
Source: Front Public Health. 2023 Apr 6;11:1151038. doi: 10.3389/fpubh.2023.1151038 (PMC10117903; doi:10.3389/fpubh.2023.1151038)
Supplement: Supplementary file 2 [file Table_2.DOC]

|  | **area Ⅰ** | **area Ⅱ** | **area Ⅲ** |
| --- | --- | --- | --- |
| outbreak phase | 598.82±91.97^＊,§^  (805.87±225.95) ^†^ | 374.93±60.08^§^  (397.80±63.02) ^†^ | 270.60±41.06 |
| phase II | 589.65±141.54 | 561.65±110.60 | 432.25±76.92 |
| phase III | 604.37±121.68^＊,§^  (933.38±349.71) ^†^ | 257.37±60.11^§^  (298.32±70.94) ^†^ | 188.47±42.25 |
| March 18 to June 15, 2020 | 0.10±0.06 | 0 | 22.16±2.57^＊＊^ |
| June 16 to July 2, 2020 | 0 | 0 | 18.88±2.61^＊＊^ |

**Supplementary material 2 The average number of confirmed COVID-19 cases increased daily in different areas of Chinese mainland during different phases and periods** area I, Wuhan. area II, Hubei province (excluding Wuhan city). Area III, Chinese mainland (excluding Hubei province). Statistical comparison after excluding the data of February 12, 2020 in area I and II. ^＊^, *P*＜0.05, ^＊＊^, *P*＜0.01, compared with the same-phase values of the other two areas.^§^, The average value obtained without including the data of this area on February 12, 2020. ^†^, The average value obtained without excluding the data of this area on February 12, 2020.
