# Supplementary material for: COVID-19 epidemic phases and morbidity in different areas of Chinese mainland, 2020
Source: Front Public Health. 2023 Apr 6;11:1151038. doi: 10.3389/fpubh.2023.1151038 (PMC10117903; doi:10.3389/fpubh.2023.1151038)
Supplement: Supplementary file 3 [file Table_3.DOC]

|  | **area Ⅰ** | **area Ⅱ** | **area Ⅲ** |
| --- | --- | --- | --- |
| outbreak phase | 16370.79±1696.19^＊^ | 7143.18±703.34^＊^ | 4581.67±456.47^＊^ |
| phase II | 2948.91±773.47 | 2841.59±750.31 | 2287.19±571.44 |
| phase III | 24286.26±1641.12^＊,§^ | 9754.86±651.42^＊,§^ | 5728.91±517.19^＊,§^ |
| March 18 to June 15, 2020 | 665.69±157.09 | 9.64±3.70^＊^ | 413.97±34.78 |
| June 16 to July 2, 2020 | 0 | 0 | 365.18±13.84^＊^ |

**Supplementary material 3 Average number of in-hospital confirmed COVID-19 cases daily in different areas of Chinese mainland during different phases and periods.** area I, Wuhan. area II, Hubei province (excluding Wuhan city). Area III, Chinese mainland (excluding Hubei province). ^＊^, *P*＜0.01, compared with the same-phase values of the other two areas. ^§^, *P*＜0.01, compared with the same-area value of phase II.
