# Supplementary material for: COVID-19 epidemic phases and morbidity in different areas of Chinese mainland, 2020
Source: Front Public Health. 2023 Apr 6;11:1151038. doi: 10.3389/fpubh.2023.1151038 (PMC10117903; doi:10.3389/fpubh.2023.1151038)
Supplement: Supplementary file 4 [file Table_4.DOC]

|  | **area Ⅰ** | **area Ⅱ** | **area Ⅲ** |
| --- | --- | --- | --- |
| April 1 to June 15, 2020 | 15.63±1.38 | 0.53±0.09^＊^ | 11.71±1.81 |
| June 16 to July 2, 2020 | 0 | 0.06±0.06 | 5.71±0.71^＊^ |

**Supplementary material 4 The average number of asymptomatic infections increased daily in different areas of Chinese mainland during different periods** area I, Wuhan. area II, Hubei province (excluding Wuhan city). Area III, Chinese mainland (excluding Hubei province). ^＊^, *P*＜0.01, compared with the same-period values of the other two areas.
