# Supplementary material for: COVID-19 epidemic phases and morbidity in different areas of Chinese mainland, 2020
Source: Front Public Health. 2023 Apr 6;11:1151038. doi: 10.3389/fpubh.2023.1151038 (PMC10117903; doi:10.3389/fpubh.2023.1151038)
Supplement: Supplementary file 5 [file Table_5.DOC]

|  | **area Ⅰ** | **area Ⅱ** | **area Ⅲ** |
| --- | --- | --- | --- |
| April 1 to June 15, 2020 | 441.16±23.98^＊^ | 14.75±1.20^＊^ | 234.92±17.07^＊^ |
| June 16 to July 2, 2020 | 0^＊^ | 2.12±0.27^＊^ | 101.53±1.30^＊^ |

**Supplementary material 5 The average number of in-hospital asymptomatic infections daily in different areas of Chinese mainland during different periods.** area I, Wuhan. area II, Hubei province (excluding Wuhan city). Area III, Chinese mainland (excluding Hubei province). ^＊^, *P*＜0.01, compared with the same-period values of the other two areas.
