# Supplementary material for: COVID-19 epidemic phases and morbidity in different areas of Chinese mainland, 2020
Source: Front Public Health. 2023 Apr 6;11:1151038. doi: 10.3389/fpubh.2023.1151038 (PMC10117903; doi:10.3389/fpubh.2023.1151038)
Supplement: Supplementary file 6 [file Table_6.DOC]

| **area Ⅰ** | **area Ⅱ** | **area Ⅲ** |
| --- | --- | --- |
| 0.52±0.24 | 0 | 131.00±19.50^＊^ |

**Supplementary material 6 The average number of confirmed COVID-19 cases increased weekly in different areas of Chinese mainland from July 3, 2020 to January 1, 2021** area I, Wuhan. area II, Hubei province (excluding Wuhan city). area III, Chinese mainland (excluding Hubei province). ^＊^, *P*＜0.01, compared with the values of the other two areas.
